# Embryonic Stem Cell Proliferation Stimulated By Altered Anabolic Metabolism From Glucose Transporter 2-Transported Glucosamine

**DOI:** 10.1038/srep28452

**Published:** 2016-06-17

**Authors:** Jin Hyuk Jung, Kumiko Iwabuchi, Zhihong Yang, Mary R. Loeken

**Affiliations:** 1 Section on Islet Cell and Regenerative Biology Joslin Diabetes Center, Boston, MA 02215, USA; 2 Department of Medicine, Harvard Medical School, Boston, MA 02115, USA; 3 Section on Vascular Cell Biology Harvard Medical School, Boston, MA 02115, USA

## Abstract

The hexose transporter, GLUT2 (SLC2A2), which is expressed by mouse embryos, is important for survival before embryonic day 10.5, but its function in embryos is unknown. GLUT2 can transport the amino sugar glucosamine (GlcN), which could increase substrate for the hexosamine biosynthetic pathway (HBSP) that produces UDP-N-acetylglucosamine for *O*-linked N-acetylglucosamine modification (*O*-GlcNAcylation) of proteins. To understand this, we employed a novel murine embryonic stem cell (ESC) line that, like mouse embryos, expresses functional GLUT2 transporters. GlcN stimulated ESC proliferation in a GLUT2-dependent fashion but did not regulate pluripotency. Stimulation of proliferation was not due to increased *O*-GlcNAcylation. Instead, GlcN decreased dependence of the HBSP on fructose-6-PO_4_ and glutamine. Consequently, glycolytic- and glutamine-derived intermediates that are needed for anabolic metabolism were increased. Thus, maternally obtained GlcN may increase substrates for biomass accumulation by embryos, as exogenous GlcN does for GLUT2-expressing ESC, and may explain the need for GLUT2 expression by embryos.

*Glut2*, also called *Slc2a2* (solute carrier family 2 (facilitated glucose transporter), member 2), mRNA is expressed by mouse embryos during pre- and early postimplantation development^1^. GLUT2 is classically known as a high K_M_ (~16 mM) glucose transporter that is expressed by pancreatic β cells and liver^2^. However, a biological function to explain why embryos express GLUT2 is not obvious. GLUT2 is not likely to function as a glucose transporter because its K_M_ for glucose is much higher than glucose concentrations in the oviduct (3.4 mM) or in maternal circulation (5.5 mM)^2^,^3^. Furthermore, embryos also express GLUT1 (SLC2A1) and GLUT3 (SLC2A3)^1^,^4^,^5^, transporters with K_M_s for glucose (~5 mM) that are equivalent to normal maternal blood glucose concentrations and so, would transport glucose more efficiently than GLUT2. However, we found that *GLUT2*^+/−^ and *GLUT2*^−/−^ embryos were not recovered on embryonic (E) 10.5 from heterozygous crosses at the expected Mendelian frequencies (ratios of *GLUT2*^+/+^: *GLUT2*^+/−^: *GLUT2*^−/−^ were 52%: 30%: 18% instead of the expected 25%: 50%: 25%)^6^. This demonstrated that GLUT2 performs an, as yet, undetermined function that is important for embryo survival prior to E 10.5.

The amino sugar, GlcN, can be transported by GLUT2 when expressed by microinjected *Xenopus* oocytes, transfected HEK293T cells, or hepatocytes, with a K_M_ of 0.8 mM^7^. GlcN can also be transported by GLUT1, but with a higher K_M_ (2 mM). However, it is not known if GLUT2 functions as a physiological GlcN transporter for any tissue.

Exogenously transported GlcN can serve as substrate for the HBSP, but GlcN-6-PO_4_, the first metabolite in the HBSP, can also be synthesized from the glycolytic intermediate, fructose-6-PO_4_, plus the amino acid, glutamine by the rate-limiting enzyme, glutamine-fructose-6-PO_4_ amidotransferase (GFAT)^8^,^9^. The HBSP generates UDP-N-Acetyl-GlcN for *N*- and *O*-linked glycosylation reactions. Many signaling proteins and transcription factors are modified by *O*-GlcNAcylation, having opposite effects on their activities from phosphorylation^10^,^11^. There are reports that *O*-GlcNAcylation of the pluripotency transcription factors, OCT4 and SOX2, is required to inhibit differentiation^12^, and that GLUT1-transported supplemental GlcN stimulates ESC proliferation utilizing signaling pathways dependent on *O*-GlcNAcylation^13^,^14^. Knockout of *Ogt*, which encodes *O*-GlcNAc transferase, the enzyme that catalyzes *O*-GlcNAcylation, is embryonic lethal^15^. Thus, transport of exogenous GlcN could increase substrate for *O*-GlcNAcylation may be important to promote embryo and ESC pluripotency and self-renewal.

Study of the function of GLUT2-mediated GlcN transport on embryo cell physiology *in vivo* is constrained by the inability to reduce or eliminate GlcN from maternal circulation, but could be circumvented, in theory, by use of embryo-derived ESC to study these processes *in vitro*. However, a shortcoming of existing ESC lines is that they (like most mammalian cell lines) are conventionally isolated and propagated in media containing a high glucose concentration (25 mM) which causes high rates of glucose influx by GLUT2-expressing pre- or postimplantation embryos, which can induce oxidative stress and cell death as shown by us and others^6^,^16^,^17^,^18^,^19^,^20^. We recently showed that D3 ESC, a long-established ESC line that was isolated and maintained in high glucose media^21^, express barely detectable levels of GLUT2 and transport glucose only with low K_M_ kinetics^22^, suggesting that ESC lines that survive isolation in high glucose media stably adapt to the adverse high glucose environment by losing expression or function of GLUT2. However, an ESC line that we established in media containing a physiological glucose concentration (5.5 mM), which we named, LG-ESC (Low Glucose-isolated ESC), expresses GLUT2 and transports glucose with the same kinetics as does the embryo^12^,^13^,^14^. Thus, LG-ESC can be used to test for a physiological role of GLUT2 as a GlcN transporter to favorably affect ESC, and by extension, embryo physiology.

## Results

### Exogenous GlcN Stimulates ESC Proliferation in a GLUT2-Dependent Manner

**Figure 1:**
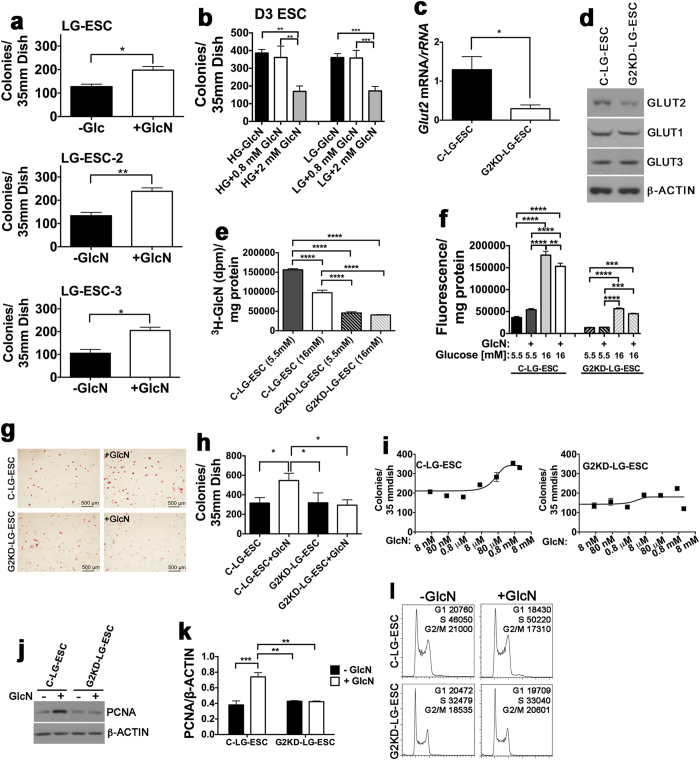
GLUT2-mediated GlcN transport stimulates ESC proliferation. (**a**) Alkaline phosphatase-positive (AP+) colonies following culture for 4 days ± 0.8 mM GlcN of 3 different LG-ESC lines (LG-ESC, LG-ESC-2, and LG-ESC-3) that were derived from 3 different FVB blastocysts in low glucose media. (**b**) AP+ D3 ESC colonies following culture for 4 days in media containing either 5 or 25 mM glucose ±0.8 or 2 mM GlcN. (**c**) RT-PCR of *Glut2* (*Slc2a2*) mRNA normalized to *rRNA* from control LG-ESC (C-LG-ESC) and *Glut2* knockdown (G2KD-LG-ESC) transfected with shRNA plasmids as described in Methods. (**d**) Immunoblot of GLUT2, GLUT1 (SLC2A1), GLUT3 (SLC2A3), and β-ACTIN from C-LG-ESC and G2KD-LG-ESC. (**e**) Transport of 0.8 mM ^3^H-GlcN by C-LG-ESC and G2KD-LG-ESC in the presence of 5.5 mM or 16 mM glucose. (**f**) Transport of 5.5 or 16 mM 2-deoxy-D-glucose containing the fluorescent 2-deoxy-D-glucose analog, 2-NBD-glucose, by C-LG-ESC and G2KD-LG-ESC ± 0.8 mM GlcN. (**g**) AP staining of C-LG-ESC and G2KD-LG-ESC cultured ± 0.8 mM GlcN. Scale bar = 500 μm. (**h**) Quantitation of AP+ colonies. (**i**) Numbers of AP+ C-LG-ESC and G2KD-LG-ESC colonies in response to 8 nM-8 mM GlcN. (8 mM GlcN was toxic to both cell lines.) (**j**) Immunoblot of PCNA from C-LG-ESC and G2KD-LG-ESC cultured ± 0.8 mM GlcN. (**k**) Quantitation of PCNA/β-ACTIN from triplicate culture dishes. (**l**) Flow cytometry analysis by C-LG-ESC and G2KD-LG-ESC cultured ± 0.8 mM GlcN. The total numbers of cells in G1, S and G2/M from three replicate columns are indicated. Experiments were repeated 2–3 times using triplicate culture dishes. Quantitative data from representative experiments are displayed as the mean ± s.e.m. (*N* = 3) and were analyzed by Student t-test (**a**,**c**) one-way ANOVA followed by Tukey’s post test (**b**,**e**,**h**,**k**), two-way ANOVA followed by Tukey’s post test (**f**), nonlinear regression (**i**) or chi-square (**l**). *P < 0.05; **P < 0.01; ***P < 0.001; ****P < 0.0001.

Because of previous reports that increased *O*-GlcNAcylation stimulates ESC proliferation or pluripotency^12^,^13^,^14^, we hypothesized that the normal function of GLUT2 for embryos and GLUT2-expressing ESC is to transport GlcN, which would increase substrate for *O*-GlcNAcylation and stimulate proliferation and/or pluripotency. To test this, we stained LG-ESC colonies for alkaline phosphatase, a marker of pluripotency^23^, after culture with or without GlcN added at 0.8 mM, the K_M_ of GLUT2 for GlcN. All colonies were alkaline phosphatase-positive (AP+), and GlcN increased numbers of AP+ LG-ESC colonies 1.6-fold (Fig. 1a). GlcN increased numbers of AP+ colonies of two additional independently-derived LG-ESC lines, LG-ESC-2 and LG-ESC-3, that also express *Glut2*^22^ by 1.8- and 2-fold, respectively. In contrast, the conventional D3 ESC, which are normally cultured in high glucose media^21^ and lack functional GLUT2 transporters^22^ were AP+ regardless of GlcN supplementation. However, GlcN did not increase numbers of AP+ D3 ESC colonies when added at 0.8 or 2 mM GlcN (which would be taken up by GLUT2 or GLUT1, respectively) (Fig. 1b). In fact, more than 2-fold fewer colonies were generated in the presence of 2 mM GlcN than in 0 or 0.8 mM GlcN. This was not due to competition for GlcN uptake by the high concentration of glucose (25 mM) in the media, because neither 0.8 nor 2 mM GlcN increased AP+ colonies in low glucose (5.5 mM) media. *Ogt* resides on the X chromosome^15^ but stimulation of AP+ colony numbers by GlcN was not correlated with *Ogt* copy numbers per genome, because LG-ESC and D3 ESC are both male, and LG-ESC-2 and LG-ESC-3 are both female (Supplementary Table 1).

To determine whether GLUT2 is required for the GlcN-stimulated increase in AP+ colonies, *Glut2* mRNA was knocked down in LG-ESC with a plasmid constitutively expressing *Glut2* shRNA. The resulting cell line was called G2KD-LG-ESC. A control cell line, transfected with empty plasmid, was called C-LG-ESC. *Glut2* mRNA was reduced more than 4-fold (Fig. 1c), and protein levels were reduced almost 2-fold (Fig. 1d). There was no effect of *Glut2* shRNA on protein levels of the low K_M_ glucose transporters, GLUT1 and GLUT3 (Fig. 1d) that are also expressed by pre- and early postimplantation embryos^1^,^4^,^5^,^12^. To obtain functional evidence that *Glut2* shRNA reduces low K_M_ GlcN/high K_M_ glucose transport activity, transport of 0.8 mM ^3^H-GlcN in the presence of 5.5 or 16 mM glucose, and transport of 5.5 or 16 mM 2-NBD-glucose (a fluorescent 2-deoxy-D-glucose analog) in the presence of 0.8 mM GlcN were assayed. ^3^H-GlcN was transported by C-LG-ESC and was inhibited 40% by 16 mM glucose, compared to 5.5 mM glucose, whereas ^3^H-GlcN transport was significantly reduced by knock down of *Glut2* mRNA and there was no effect of the concentration of co-incubated glucose (Fig. 1e). Transport of 2-NBD-glucose by C-LG-ESC was significantly increased at 16 mM 2-deoxy-D-glucose compared to 5.5 mM 2-deoxy-D-glucose, and 16 mM 2-NBD-glucose uptake was inhibited 16% by GlcN (Fig. 1f). Although 5.5 and 16 mM 2-NBD-glucose uptake was reduced in G2KD-LG-ESC, there was no significant effect of GlcN on uptake of either 5.5 or 16 mM 2-NBD-glucose. These results demonstrate that GLUT2 expressed by C-LG-ESC transports GlcN and 16 mM glucose with characteristics of a low K_M_ GlcN/high K_M_ glucose transporter and that C-LG-ESC and G2KD-LG-ESC can be used to study GLUT2-dependent effects of GlcN on ESC.

To investigate whether stimulation of increased numbers of AP+ colonies by GlcN is GLUT2-dependent, C-LG-ESC and G2KD-LG-ESC were cultured ± GlcN and then stained for AP. C-LG-ESC colonies were increased 1.7-fold by GlcN, but there was no effect of GlcN on numbers of AP+ G2KD-LG-ESC colonies (Fig. 1g,h). To determine the dose-dependence on GlcN to increase colony numbers, cultures were incubated with 0.8 nM to 8 mM GlcN. In cultures of C-LG-ESC, the EC_50_ was 0.04 mM, and the maximum effect occurred with 0.8 mM, the K_M_ of GLUT2 for GlcN (Fig. 1i). There was no further increase in AP+ colonies with 2 mM GlcN, the K_M_ of GLUT1 for GlcN. GlcN did not increase AP+ G2KD-LG-ESC colony numbers at any concentration. Because the increase in colony numbers suggested that GlcN stimulates proliferation, we assayed proliferating cell nuclear antigen (PCNA), a marker of DNA synthesis, by Western blot, and performed cell sorting to determine the proportion of cells in different phases of the cell cycle. GlcN increased steady state levels of PCNA 2-fold in control LG-ESC, but had no effect in G2KD-LG-ESC (Fig. 1j,k). There were significant differences in the distribution of C-LG-ESC in different stages of the cell cycle cultured + GlcN (P < 0.0001), with an increased proportion of cells in S phase and a decreased proportion of cells in G1 and G2/M (Fig. 1l). There were also significant differences in the distribution of G2KD-LG-ESC in different stages of the cells cycle that were cultured ± GlcN (P < 0.001) which was mostly due to an increase of cells in G2/M. Taken together, the increases in AP+ colony numbers, PCNA levels, and changes in cell cycle distribution, indicate that GlcN transported by GLUT2 stimulates proliferation of pluripotent ESC.

### Exogenous GlcN Does Not Increase *O*-GlcNAcylation of Core Pluripotency Transcription Factors and Does Not Inhibit Differentiation

**Figure 2:**
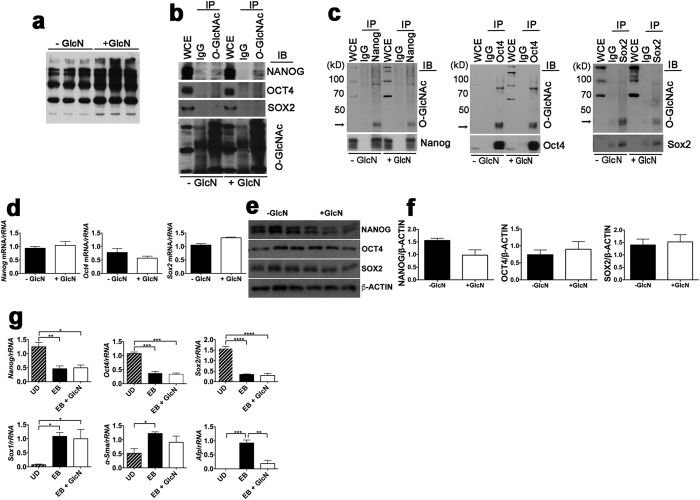
Effects of GlcN on *O*-GlcNAcylation and expression of core pluripotency factors and pluripotency of LG-ESC. (**a**) Immunoblot of total *O*-GlcNAcylated proteins from LG-ESC grown ± 0.8 mM GlcN. (**b**) Immunoblot of NANOG, OCT4 and SOX2 from whole cell extracts (WCE) of LG-ESC, or after immunoprecipitation using nonimmune mouse IgG or *O*-GlcNAc antibody. (**c**) Immunoblot of *O*-GlcNAcylated proteins in whole cell extracts, or after immunoprecipitation using nonimmune rabbit IgG or antibodies against NANOG, OCT4 or SOX2. Positions of NANOG, OCT4, and SOX2 are indicated by arrows. (**d**) RT-PCR of *Nanog*, *Oct4* and *Sox2* mRNA normalized to *rRNA* from LG-ESC cultured ± 0.8 mM GlcN. (**e**) Immunoblot of NANOG, OCT4, SOX2, or β-ACTIN from LG-ESC. (**f**) Quantitation of (**e)**. (**g**) RT-PCR of *Nanog*, *Oct4*, *Sox2, Sox1*, α-*Sma*, or *Afp* mRNA normalized to *rRNA* from undifferentiated (UD) or embryoid bodies (EB) LG-ESC cultured ± 0.8 mM GlcN. Experiments were repeated 2–3 times using triplicate culture dishes. Data from representative experiments are displayed as the mean ± s.e.m. (*N* = 3) and were analyzed by Student t-test (**d**,**f**), or one-way ANOVA followed by Tukey’s post test (**g**). *P < 0.05; **P < 0.01; ***P < 0.001; ****P < 0.0001.

We considered that stimulation of LG-ESC proliferation by exogenous GlcN was due to stimulation of self-renewal of pluripotent ESC caused by increased *O*-GlcNAcylation of the pluripotency transcription factors, OCT4 or SOX2. To investigate this, we examined *O*-GlcNAcylation levels of OCT4 and SOX2, as well as NANOG (which was previously not found to be *O*-GlcNAcylated^12^) after culture ±GlcN. GlcN increased *O*-GlcNAcylation of total LG-ESC proteins (Fig. 2a). Unexpectedly, immunoprecipitation of total *O*-GlcNAcylated proteins indicated that NANOG was *O*-GlcNAcylated, but that OCT4 or SOX2 were not, and that there was no effect of culture with GlcN (Fig. 2b). However, immunoprecipitation of each of the pluripotency factors indicated that all three are *O*-GlcNAcylated, but that GlcN did not increase their *O*-GlcNAcylation (Fig. 2c). GlcN had no significant effect on mRNA (Fig. 2d) or protein (Fig. 2e,f) levels of any of the core pluripotency transcription factors.

The lack of effect of GlcN on either steady state levels or posttranslational modification of the core pluripotency transcription factors in LG-ESC could indicate that GlcN does not stimulate pluripotency or self-renewal in these cells, but only stimulates proliferation. If this is the case, we hypothesized that GlcN would not suppress loss of pluripotency upon differentiation, nor inhibit induction of differentiation. To test this, we examined expression of *Nanog*, *Oct4*, and *Sox2*, and markers of each of the three germ layers, by embryoid bodies (EB) formed from LG-ESC cultured ± GlcN. Culture of undifferentiated LG-ESC with GlcN and during formation of EB did not inhibit the decline in expression of *Nanog*, *Oct4*, or *Sox2* by EB and did not block induction of the differentiation markers *Sox1*, α-*Sma* (Smooth muscle actin), or *Afp* (α-fetoprotein) (Fig. 2g). However, expression of α-*Sma*, a marker of mesoderm, was nonsignificantly reduced, and *Afp*, a marker of endoderm, was significantly reduced, by EB that had been cultured with GlcN. These results suggest that GlcN does not regulate maintenance or loss of pluripotency by LG-ESC, but may influence lineage fate, perhaps by stimulating proliferation of cells adopting a neuroepithelial fate more than of cells adopting mesodermal or endodermal fates.

### Increased *O*-GlcNAcylation Is Not Responsible for Stimulation of Proliferation by Exogenous GlcN

**Figure 3:**
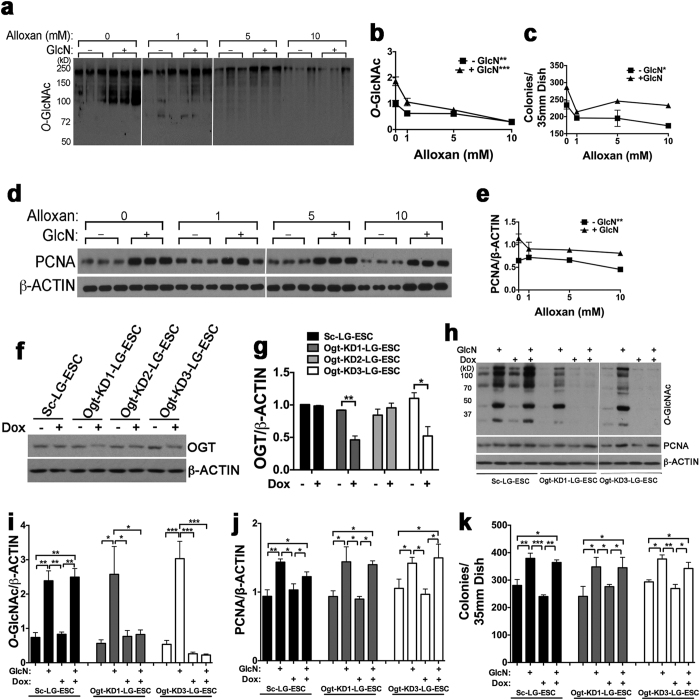
Effects of *O*-GlcNAcylation inhibition on LG-ESC proliferation. (**a**) Immunoblot of total *O*-GlcNAcylated proteins from LG-ESC grown ± 0.8 mM GlcN and 0, 1, 5 or 10 mM alloxan. (**b**) Quantitation of (**a)**. (**c**) Quantitation of AP+ LG-ESC colonies following culture as in **a**. (**d**) Immunoblot of PCNA and β-ACTIN following culture as in (**a)**. (**e**) Quantitation of (**d)**. (**f**) Immunoblot of *O*-GlcNAc transferase (OGT) from clones of LG-ESC stably transfected with doxycycline (Dox)-inducible pSingle containing a scrambled shRNA sequence (Sc-LG-ESC) or one of three different shRNA sequence targeting *Ogt* mRNA (Ogt-KD1-, Ogt-KD2-, or Ogt-KD3-LG-ESC), cultured ± 1 μg/ml Dox. (**g**) Quantitation of OGT/β-ACTIN assayed as in (**f)**. (**h**) Immunoblot of total *O*-GlcNAcylated proteins, PCNA, and β-ACTIN from Sc-LG-ESC, Ogt-KD1-LG-ESC, or Ogt-KD3-LG-ESC treated ± 1 μg/ml Dox and ± 0.8 mM GlcN. (**i**) Quantitation of *O*-GlcNAcylated proteins from Sc-LG-ESC, Ogt-KD1-LG-ESC, or Ogt-KD3-LG-ESC assayed as in (**h)**. (**j**) Quantitation of PCNA from Sc-LG-ESC, Ogt-KD1-LG-ESC, or Ogt-KD3-LG-ESC assayed as in (**h)**. (**k**) Quantitation of AP+ colonies of Sc-LG-ESC, Ogt-KD1-LG-ESC, or Ogt-KD3-LG-ESC cultured ± 1 μg/ml Dox and ± 0.8 mM GlcN. Experiments were repeated 2–3 times using triplicate culture dishes. Data from representative experiments are displayed as the mean ± s.e.m. (*N* = 3) and were analyzed by linear regression (**b**,**c**, and **e**), Student’s t test (**g**) or two-way ANOVA followed by Tukey’s post test of each clone (**i**–**k**). *P < 0.05, **P < 0.01, ***P < 0.001.

Although we did not observe increased *O*-GlcNAcylation of any of the core pluripotency transcription factors by exogenous GlcN, it is possible that increased *O*-GlcNAcylation of other proteins is responsible for stimulation of LG-ESC proliferation by exogenous GlcN. To investigate this, we used two different approaches to inhibit OGT activity. First, we treated LG-ESC with 1–10 mM of the OGT inhibitor, alloxan^24^. Alloxan inhibited basal as well as GlcN-stimulated global protein *O*-GlcNAcylation in a dose-dependent fashion (Fig. 3a,b). However, while alloxan reduced numbers of AP+ colonies and PCNA levels in the absence of GlcN, only 1 mM alloxan inhibited the GlcN-stimulated increase in AP+ colonies and PCNA levels, and there was no further inhibition of GlcN-stimulation of AP+ colonies and PCNA levels by 5 and 10 mM alloxan (Fig. 3c–e). This suggests that *O*-GlcNAcylation dependent on fructose 6-PO_4_ and glutamine is necessary for proliferation, but that further stimulation of *O*-GlcNAcylation by GlcN does not further stimulate proliferation.

There could be effects of alloxan independent of OGT inhibition, for example, reduced GLUT2-mediated solute transport^25^, and alloxan may be unstable at physiological pH and temperature^26^. This could explain the lack of a dose-dependent effect of alloxan on GlcN-stimulated increase in AP+ colonies and PCNA levels. Therefore, we tested the effect of suppressing *Ogt* mRNA with shRNA. LG-ESC were stably transfected with a tetracycline-inducible plasmid containing one of three different *Ogt* shRNA sequences. Two of the *Ogt* shRNA sequences (shRNA1 and -3) reduced OGT protein by 2-fold after doxycycline (Dox) treatment, but there was no effect of a scrambled shRNA sequence (Fig. 3f,g). Dox inhibited GlcN-stimulated total protein *O*-GlcNAcylation by *Ogt*-KD1- and *Ogt*-KD-3-LG-ESC up to 13-fold (Fig. 3h,i) but did not inhibit either the fructose 6-PO_4_ and glutamine-dependent or the GlcN-stimulated increase in PCNA levels (Fig. 3h,j) or numbers of AP+ colonies (Fig. 3k). These results suggest that, while GLUT2-mediated GlcN transport provides more substrate for *O*-GlcNAcylation, increased *O*-GlcNAcylation is not responsible for stimulation of LG-ESC proliferation by GlcN.

### GlcN Stimulates Anabolic Metabolic Pathways by Shifting Fructose 6-PO_4_ and Glutamine from the HBSP Without Changing Net Aerobic Metabolism

**Figure 4:**
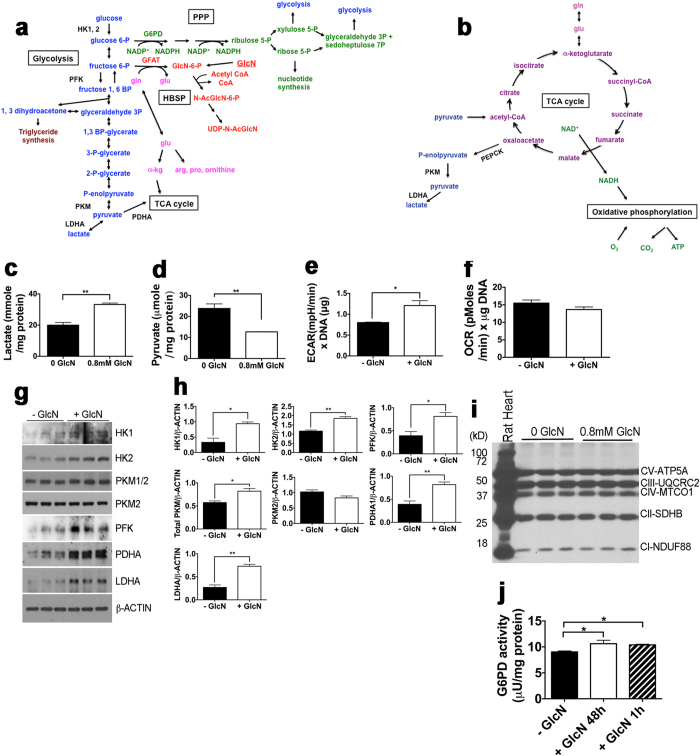
GlcN effects on glucose- and glutamine-utilizing metabolic pathways. (**a**) Schematic diagram of the hexosamine biosynthetic (HBSP, red), glycolytic (blue), pentose phosphate (PPP, green), and glutamine (gln, pink) -derived pathways. GlcN 6-P, the first intermediate in the HBSP, can be generated upon phosphorylation of exogenous GlcN (underlined), or from fructose 6-P + gln. Hexokinase-1 and -2 (HK1, 2), phosphofructokinase (PFK), pyruvate kinase M (PKM), pyruvate dehydrogenase (PDHA), lactate dehydrogenase (LDHA), glucose 6-PO_4_ dehydrogenase (G6PD), Glutamine--fructose-6-phosphate aminotransferase (GFAT), tricarboxylic acid cycle (TCA cycle). (**b**) Schematic diagram of oxidative pathways, the TCA cycle (purple) and oxidative phosphorylation (green), related to glucose or glutamine metabolism. (**c**) Lactate accumulation in media after 48 hr culture of LG-ESC ± 0.8 mM GlcN. (**d**) Intracellular pyruvate accumulation after 48 hr culture or LG-ESC ± 0.8 mM GlcN. (**e**) Extracellular acidification rate (ECAR) by LG-ESC incubated ± 0.8 mM GlcN. (**f**) Oxygen consumption rate (OCR) by LG-ESC incubated ± 0.8 mM GlcN. (**g**) Immunoblot of glycolytic enzymes from LG-ESC cultured ± 0.8 mM GlcN. (**h**) Quantitation of (**g)**. (**i**) Immunoblot of subunits of mitochondrial respiratory chain enzymes (Complex V ATP synthase (CV-ATP5A), Complex III ubiquinol-cytochrome c reductase (CIII-UQCRC2), Complex IV cyctochrome C oxidase catalytic (CIV-MTCO1), Complex II succinate dehydrogenase iron-sulfur protein (CII-SDHB) and Complex I NADH dehydrogenase (CI-NDUFB8)) from rat heart (positive control) or extracts from LG-ESC cultured ± 0.8 mM GlcN. (**j**) G6PD activity by LG-ESC grown ± 0.8 mM GlcN for 48 hr or +GlcN only the last hr of culture. Experiments were repeated 2–3 times using triplicate culture dishes. Quantitative data from representative experiments are displayed as the mean ± s.e.m. (*N* = 3) and were analyzed by Student t-test *P < 0.05, **P < 0.01.

Based on the results presented above, we hypothesized that increased concentrations of GlcN 6-PO_4_ from transported GlcN reduces the flux of fructose 6-PO_4_ and glutamine into the HBSP and would shift metabolites toward glycolysis, the pentose phosphate pathway (PPP), and glutaminolysis for the tricarboxylic acid (TCA) cycle and energy production and/or protein and nucleic acid synthesis (Fig. 4a,b). This suggests that increased substrates for growth of the biomass are the stimuli for proliferation in response to exogenous GlcN, rather than signaling by increased *O*-GlcNAcylated proteins.

We studied the effects of GlcN on glucose-and glutamine-dependent metabolism using several approaches. Assay of lactate accumulation in media and pyruvate accumulation intracellularly demonstrated that GlcN significantly increased lactate production and inhibited pyruvate levels (Fig. 4c,d). The decreased pyruvate accumulation could not explain the increased lactate production, because approximately 1000-fold more lactate than pyruvate was generated both in the presence and absence of GlcN. Extracellular flux analysis demonstrated that GlcN increased the extracellular acidification rate (ECAR), an indicator of the rate of lactate production (Fig. 4e) but had no effect on the oxygen consumption rate (OCR) (Fig. 4f). The increases in lactate accumulation and ECAR suggest that GlcN increases glycolytic flux. The decrease in pyruvate levels with no change in OCR could be due to increased utilization of TCA cycle intermediates for biosynthetic reactions, such as conversion of pyruvate to Acetyl CoA for the HBSP, with increased anaplerosis of the TCA cycle by glutamine (via glutamate) to maintain energy production. Immunoblot of glycolytic enzymes indicated that GlcN increased steady state levels of several glycolytic enzymes 1.4 to 3-fold, including pyruvate dehydrogenase, which regulates entry of pyruvate into the TCA cycle (Fig. 4g,h). However, immunoblot of mitochondrial enzymes demonstrated that steady state levels of enzymes that regulate oxidative phosphorylation were unchanged (Fig. 4i). Assay of glucose 6-PO_4_ dehydrogenase (G6PD), the rate-limiting enzyme of the PPP, showed that GlcN stimulated G6PD activity, and this effect occurred within 1 hour, suggesting that stimulation of G6PD activity by GlcN does not require protein synthesis (Fig. 4j). Because GlcN 6-PO_4_ competes with glucose 6-PO_4_ for binding to G6PD^27^, stimulation of G6PD by GlcN suggests that GlcN increases G6PD substrate (glucose 6-PO_4_) accumulation.

**Figure 5:**
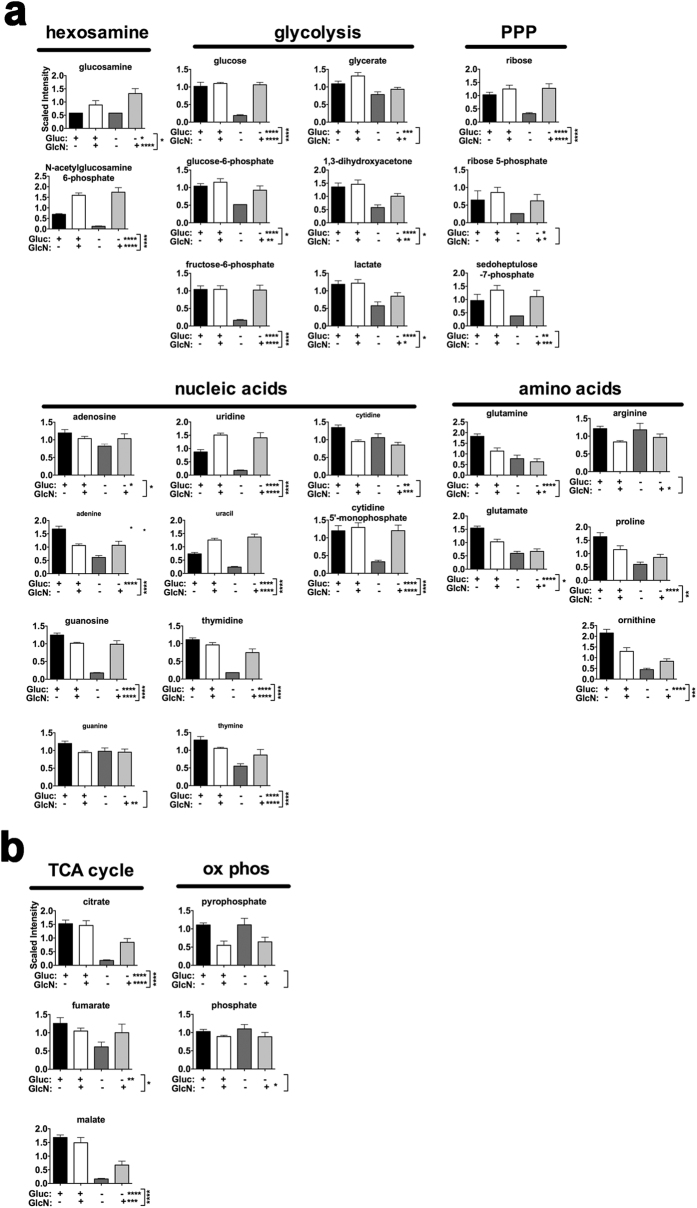
GlcN effects on metabolic profiles. (**a**) Metabolites of anabolic pathways supplied by glucose or glutamine. (**b**) Metabolites of energy production (TCA cycle or oxidative phosphorylation) fueled by glucose or glutamine. LG-ESC were grown in low glucose media ± 0.8 mM GlcN for 47 h, and then half of the dishes were replaced with the same media and half were replaced with 0 glucose media ± 0.8 mM GlcN (24 plates total, 6 per treatment group) for 1 additional hr of culture. Methods for metabolic profiling, metabolite identification, and data analysis are described in the Supplementary Methods. Statistical analysis of log-transformed data was performed using “R” (http://cran.r-project.org/). Data (mean ± s.e.m., *N* = 6) were analyzed by two-way ANOVA to determine main effects of glucose (Gluc), GlcN, and Gluc:GlcN interaction. Multiple comparisons were accounted for by estimating the false discovery rate (FDR) using q-values. *q < 0.05, **q < 0.01, ***q < 0.001, ****q < 0.0001. (q values of Gluc:GlcN interactions are shown next to vertical brackets.) Detailed quantitation and results of statistical analyses are shown in Supplementary Table 2.

To determine whether GlcN altered metabolic utilization of glucose or glutamine, or both, LG-ESC were grown for 47 hours in low glucose media ± GlcN, then in glucose-containing or glucose-depleted media, ± GlcN, for a final hour of culture, and then untargeted metabolic profiling of steady state levels of several metabolites in glucose- and glutamine-utilizing pathways was performed. Scaled intensity levels of several intermediates in the HSBP, glycolysis, the PPP, and nucleic acids and amino acids (Fig. 5a), as well as intermediates in the TCA cycle and oxidative phosphorylation (Fig. 5b) showed that there are significant main effects of glucose removal or of glucosamine presence or absence or significant interactions between glucose removal and presence or absence of glucosamine for several metabolites. ANOVA contrasts of the metabolite profiles (Supplementary Table 2) demonstrated significant increases in many metabolites for glycolysis, the PPP, the TCA cycle, and nucleotides in glucose-withdrawn cells if they were cultured with GlcN ((−)GLU(+)GLCN/(−)GLU(−)GLCN)). This indicates that GlcN causes a shift in glucose-dependent metabolites (including those upstream of fructose 6-PO_4_) that remains even after glucose removal. Furthermore, there were very few significant changes (which, except for GlcN, were all decreases) in metabolites (mostly glutamine-derived amino acids, and glycolytic and TCA cycle intermediates) in cells cultured with GlcN after glucose removal compared to those maintained in glucose plus GlcN ((−)GLU(+)GLCN/(+)GLU(+)GLCN). Because many metabolites do not change in GlcN-treated cultures after acute glucose withdrawal, this indicates that addition of GlcN decreases glucose entry into the HBSP and increases glucose-derived metabolites, and this effect remains even after glucose removal. Comparison of glutamine-derived metabolites and TCA cycle intermediates in the presence or absence of GlcN, with or without glucose removal, suggests that GlcN also alters the fate of glutamine. Decreases in glutamine-derived amino acids, as well as in most purines and pyrimidines, could be due to increased incorporation of these substrates into proteins and DNA. However, the maintenance of TCA cycle intermediates in the absence of glucose suggests that GlcN also stimulates anaplerosis of the TCA cycle by glutamine.

## Discussion

**Figure 6:**
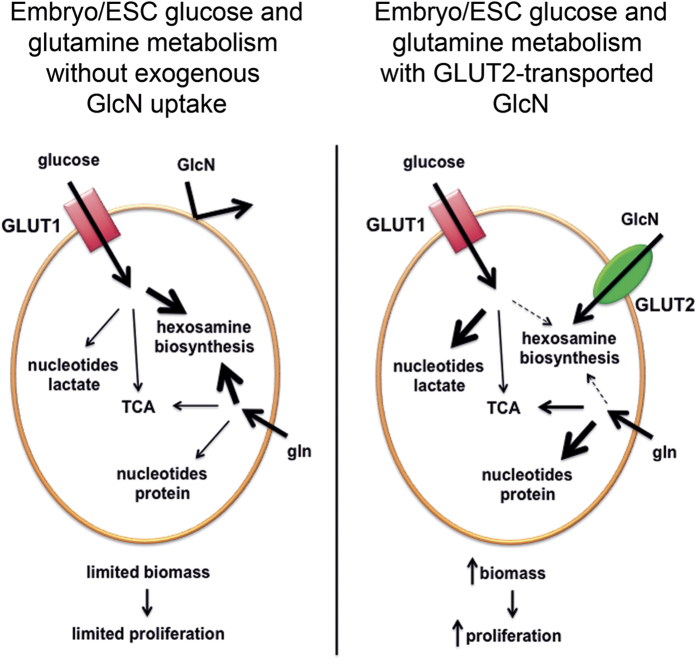
Effect of GLUT2-transported GlcN on glucose and glutamine utilization. In the absence of exogenous GlcN or GLUT2 to transport GlcN (left panel), the HBSP must be supported by glucose + glutamine, but at the expense of pathways that support biomass accumulation. In the presence of GLUT2-transported GlcN, GlcN supports the HBSP, thereby increasing availability of glucose and glutamine for pathways that support biomass accumulation.

It has long been known that the HBSP is active and important very early in embryonic development because inhibiting *N*-linked glycosylation with tunicamycin inhibits blastocyst formation^28^,^29^, and ESC and mouse embryos lacking OGT are not viable^15^. However, the importance of transport of exogenous (e.g., maternally-generated GlcN) substrate for the HBSP, instead of synthesis from fructose-6-PO_4_ and glutamine, has not previously been appreciated. GlcN is primarily synthesized in the liver, and the free GlcN in plasma under unsupplemented conditions is estimated to be 60–80 μM^30^,^31^, slightly higher than the EC_50_ for GlcN-stimulated increase in LG-ESC colonies. The findings presented here, using LG-ESC that express functional GLUT2 transporters as an *in vitro* model to study the function of GLUT2 expressed by early embryos, indicate that in the absence of GLUT2-stimulated GlcN uptake, the HBSP is solely dependent on fructose-6-PO_4_ and glutamine, but as a consequence, there is less availability of substrates for proliferation and biomass accumulation. This suggests that GlcN obtained from maternal circulation is an essential nutrient for embryos because embryo cells are unable to synthesize sufficient amounts of GlcN 6-PO_4_ from fructose 6-PO_4_ and glutamine without compromising glucose- and glutamine-derived metabolites for macromolecular synthesis and energy production. A schematic diagram of the differential utilization of glucose and glutamine in the absence and presence of GLUT2-transported GlcN is shown in Fig. 6.

Our findings suggest that insufficient substrate for biomass accumulation as well as to generate ATP to support growth may explain the survival disadvantage of *Glut2*^+/−^ and *Glut2*^−/−^ embryos^6^. It remains to be tested whether supplemental administration of GlcN to pregnant dams can rescue GLUT2-deficient embryos. However, high concentrations of GlcN would be transported via GLUT1, potentially competing with glucose for transport. Consequently, while GLUT2-transported GlcN increased glucose 6-PO_4_ levels, GLUT1-transported GlcN might increase the GlcN 6-PO_4_: glucose 6-PO_4_ ratio, leading to inhibition of the PPP^27^. If this happens, supplemental GlcN could mimic the adverse effects of maternal diabetes, as has been shown by others and us^32^,^33^,^34^,^35^, through inhibition of NADPH production. Therefore, there may be a narrow protective range of GlcN concentrations if it can rescue GLUT2-deficient embryos.

Our findings differ from those of some groups that reported that *O*-GlcNAcylation of pluripotency or cell cycle regulatory factors stimulates proliferation or pluripotency and inhibits differentiation of ESC^12^,^13^,^14^,^36^. We found that exogenous GlcN only stimulated proliferation of LG-ESC and did not stimulate pluripotency nor inhibit differentiation, and, while GlcN did increase total protein *O*-GlcNAcylation, stimulation of proliferation was not due to increased *O*-GlcNAcylation. These discrepancies can be explained by the differences in cell lines and culture conditions employed between ours and the other studies. The other groups used E14 (or E14-derived) ESC that, like D3 ESC, were isolated and studied in high glucose media and had presumably adapted to survival in a high glucose environment by loss of functional GLUT2 transporters. In addition, the other studies used higher GlcN concentrations (2 mM), which would be taken up via GLUT1. Therefore, the stoichiometry of glucose and GlcN transport and metabolism in the studies using E14 ESC would differ from LG-ESC, as well as from normal embryos. On the other hand, we observed no effect of GlcN to increase AP+ colony formation by D3 ESC and inhibition when we used 2 mM GlcN. Thus, a physiological effect of GlcN on embryo cell metabolism cannot be studied using ESC that lack functional GLUT2 transporters.

Although increased *O*-GlcNAcylation did not explain GlcN stimulation of proliferation, measurements of lactate and pyruvate accumulation, extracellular flux, G6PD activity, steady state levels of glycolytic and mitochondrial enzymes, and metabolic profiling, all indicated that exogenous GlcN shifts glucose and glutamine from the HBSP and increases substrate for glycolysis and the PPP, while maintaining capacity for energy production. While the mammalian embryo synthesizes macromolecules for new plasma membranes, proteins, and nucleic acids before implantation, it does not accumulate biomass until after implantation. Thus, the postimplantation embryo, which undergoes high rates of glucose consumption and glycolysis, may require GlcN for growth, particularly in the most rapidly proliferating regions such as the head and viscera^5^,^37^,^38^. Although our studies were performed using undifferentiated ESC, like postimplantation embryo cells, they are highly proliferative. Thus, GLUT2-transported GlcN may permit ESC and embryo cells to reach their full proliferative potential. Interestingly, GLUT2 protein levels are higher in neuronal precursors formed from LG-ESC than in undifferentiated LG-ESC^22^, and *Glut2* mRNA is expressed at high levels by E 7.5 and E 8.5 postimplantation embryos^1^,^6^. There have been recent reports of association of neural tube defects with specific allelic variants of human *GLUT2*^39^,^40^ and that *GLUT2* morpholinos cause defective brain development in zebrafish^41^. Embryonic neuroepithelial cells are still multipotent and undergo significant proliferation during neural tube formation. We observed that GlcN neither inhibited the loss of expression of pluripotency factors upon differentiation nor inhibited differentiation, but it did appear to influence cell lineage commitment. Therefore, GLUT2-dependent GlcN uptake may be crucial to support adequate growth of progenitors of highly proliferative tissue lineages.

GlcN is not a component of media that are normally used to culture human or murine ESC or iPSC (except that contributed by serum if used), and so, cells cultured without GlcN depend on glucose and glutamine in the media to synthesize intermediates for the HBSP. It remains to be determined whether human ESC or iPSC would express GLUT2 and be able to utilize exogenous GlcN to enhance proliferation and neuronal lineage development if they were isolated in low glucose media. However, we can speculate that human stem cells that express functional GLUT2 transporters might be more robust and have enhanced engraphment potential if they can take advantage of GlcN in the transplantation site milieu.

## Methods

### Cell Culture

Murine LG-ESC^22^ were cultured in low glucose (5.5 mM) DMEM containing 15% fetal calf serum (Atlanta Biologicals) as described^22^ ± GlcN (Sigma), added at 0.8 mM unless otherwise indicated. Additional cell culture details and generation of LG-ESC carrying shRNA plasmids are in the Supplementary Methods.

### GlcN and 2-deoxy-D-Glucose Transport Assays

^3^H-GlcN and fluorescent 2-deoxy-D-glucose transport were measured as described^7^,^22^ with modifications as described in the Supplementary Methods.

### Alkaline Phosphatase Staining

Cells were cultured for 4 days, then colonies were stained for AP using Fast Red TR salt and Naphthol (both from Sigma) as described^42^, except that cells were first fixed with 3.7% formaldehyde in phosphate buffered saline (PBS). Colonies in culture dishes were imaged using a Nikon SMZ800 microscope, and images were captured with a SPOT digital camera (Model 2.2.1, Diagnostic Instruments, Inc.) and processed using Adobe Photoshop CS5.1 software.

### Cell Cycle Analysis by FACS

3 × 10^5^ cells were cultured for 72 hr in 60 mm dishes, then were trypsinized and stored at −20 °C in 70% ethanol:30% PBS. Cells were resuspended in 500 μl of propidium iodide staining solution (0.5% Triton-X 100 (Sigma), 1 μg/ml PI (Cayman) and 100 μg/ml RNase (Sigma), in PBS) and 30,000 cells with a flow rate of ~100 cell/sec were measured using LSR II (Becton Dickenson). Phases of cell cycles were analyzed by FlowJo software.

### Immunoblot and Immunoprecipitation

Thirty μg of whole cell protein extracts were prepared after 4 days of culture and analyzed by immunoblot as described^43^,^44^. Sources and working dilutions of antibodies used for immunoblot are shown in Supplementary Table 3. Immunoprecipitation was performed using 500 μg of cell extract as described^44^. Sources and working dilutions of antibodies for immunoprecipitation are shown in Supplementary Table 4. Immunoblot secondary antibodies were detected by Western Lightning *Plus*-ECL (PerkinElmer) and exposure to X-ray film. Band intensities were quantitated using Adobe Photoshop CS5.1 software.

### Real Time RT-PCR

Reverse transcription-PCR was performed as described^18^ and in the Supplementary Methods.

### Lactate and Pyruvate Assays

Extracellular lactate and intracellular pyruvate accumulation after 48 hr of culture were measured using kits from Cayman Chemical according to the manufacture’s instructions.

### Extracellular Flux (XF) Analyses

Extracellular acidification rates (ECAR) and oxygen consumption rates (OCR) were analyzed using a Seahorse Extracellular Flux Analyzer (XF24) as described in the Supplementary Methods.

### G6PD Activity Assay

Cells were cultured in low glucose media for 48 hr ± 0.8 mM GlcN or with GlcN added only the last 1 hr of culture. Cells were lysed in a 5% NP-40 lysis buffer, and G6PD activity was measured spectrophotometrically as described^32^,^45^.

### Metabolomics Profiling

Cells were cultured in low glucose media ± 0.8 mM GlcN for 47 hr, then media were replaced with the same media or 0 glucose media ± 0.8 mM GlcN for the final hr of culture. Samples were prepared and metabolites were profiled by Metabolon, Inc. as described in the Supplementary Methods.

### Statistical Analyses

Data were analyzed by two-tailed Student t-test, one- or two-way analysis of variance (ANOVA) followed by Tukey’s multiple comparisons post test, chi-square, or nonlinear regression, using GraphPad Prism software v.6. Cultures were repeated 2-3 times using 3 replicate culture dishes (for 85–95% power using GraphPad StatMate). Metabolomic data were analyzed by two-way ANOVA as described further in the Supplementary Methods. P < 0.05 was considered statistically significant.

## Additional Information

**How to cite this article**: Jung, J. H. *et al*. Embryonic Stem Cell Proliferation Stimulated By Altered Anabolic Metabolism From Glucose Transporter 2-Transported Glucosamine. *Sci. Rep*. **6**, 28452; doi: 10.1038/srep28452 (2016).

## Supplementary Material

Supplementary Information
