# Supplementary material for: Embryonic Stem Cell Proliferation Stimulated By Altered Anabolic Metabolism From Glucose Transporter 2-Transported Glucosamine
Source: Sci Rep. 2016 Jun 17;6:28452. doi: 10.1038/srep28452 (PMC4911601; doi:10.1038/srep28452)
Supplement: Supplementary Information [file srep28452-s1.pdf]

SUPPLEMENTARY INFORMATION

Embryonic Stem Cell Proliferation Stimulated By Altered Anabolic Metabolism From Glucose Transporter 2-Transported Glucosamine

Jin Hyuk Jung, Kumiko Iwabuchi, Zhihong Yang, and Mary R. Loeken

List of Supplementary Materials

Supplementary Tables

Supplementary Table 1, related to Figure 1

Supplementary Table 2, related to Figure 5

Supplementary Table 3, related to Methods

Supplementary Table 4, related to Methods

Supplementary Methods

Supplementary References

Supplementary Table 1 (Related to Figure 1)

Y and Autosomal Genotype Results

**Cell line**	***Zfy***	***Glut2***
LG-ESC	+	+
LG-ESC-2	-	+
LG-ESC-3	-	+
D3 ESC	+	+

SUPPLEMENTARY DATA

Supplementary Table 2 (Related to Figure 5)

Heat map of statistically significant biochemicals profiled in this study.

Comparison mean values significantly different: red = $p \leq 0.05$, fold of change ≥ 1.00; dark green = $p \leq 0.05$, fold of change < 1.00

Comparison mean value difference approaching significance: pink = $0.05 < p < 0.10$, fold of change ≥ 1.00; light green = $0.05 < p < 0.10$, fold of change < 1.00

Blue shaded cells indicate significant ($p \leq 0.05$) ANOVA effect.

Data normalization: Bradford protein concentration

Pathway Sort Order	Super Pathway	Sub Pathway	Biochemical Name	Platform	Comp ID	KEGG	HMDB	ANOVA Contrasts – Fold of Change: (+)GLU(+)GLCN / (+)GLU(-)GLCN	(-)GLU(+)GLCN / (-)GLU(-)GLCN	(-)GLU(-)GLCN / (+)GLU(-)GLCN	(-)GLU(+)GLCN / (+)GLU(+)GLCN	Two-Way ANOVA: Glucose Main Effect	Glucosamine Main Effect	Glucose: Glucosamine Interaction
36		Glutamate Metabolism	glutamate	LC/MS pos	57	C00025	HMDB00148	0.66	1.12	0.39	0.65			
37			glutamine	LC/MS pos	53	C00064	HMDB00641	0.62	0.81	0.42	0.56			
296	Amino Acid		arginine	LC/MS pos	1638	C00062	HMDB00517	0.69	0.82	0.97	1.15			
298		Urea cycle; Arginine and Proline Metab	ornithine	GC/MS	1493	C00077	HMDB03374	0.60	1.88	0.21	0.64			
299			proline	LC/MS pos	1898	C00148	HMDB00162	0.70	1.44	0.37	0.75			
731			glucose	GC/MS	38507	C00031	HMDB00122	1.08	5.62	0.19	0.97			
732			glucose-6-phosph	GC/MS	31260	C00668	HMDB01401	1.11	1.79	0.50	0.80			
735		Glycolysis, Gluconeogenesis, and Pyru	fructose-6-phosph	GC/MS	12021	C05345	HMDB00124	1.00	6.22	0.16	0.98			
742			1,3-dihydroxyacet	GC/MS	35963	C00184	HMDB01882	1.08	1.75	0.43	0.69			
748			lactate	GC/MS	527	C00186	HMDB00190	1.03	1.47	0.49	0.70			
751	Carbohydrate		glycerate	GC/MS	1572	C00258	HMDB00139	1.21	1.19	0.72	0.71			
756		Pentose Phosphate Pathway	ribose 5-phosphat	GC/MS	561	C00117	HMDB00618	1.33	2.40	0.40	0.72			
759			sedoheptulose-7-p	GC/MS	35649	C05382	HMDB01068	1.41	2.93	0.39	0.82			
765		Pentose Metabolism	ribose	GC/MS	12080	C00121	HMDB00283	1.21	4.00	0.31	1.02			
873		Aminosugar Metabolism	glucosamine	GC/MS	18534	C00329	HMDB01514	1.53	2.28	1.00	1.49			
883			N-acetylglucosam	LC/MS neg	15107	C00357	HMDB02817	2.30	13.80	0.18	1.09			
908			citrate	GC/MS	1564	C00158	HMDB00094	0.96	4.70	0.12	0.58			
917		TCA Cycle	fumarate	GC/MS	1643	C00122	HMDB00134	0.83	1.63	0.49	0.95			
918	Energy		malate	GC/MS	1303	C00149	HMDB00156	0.89	4.07	0.10	0.45			
928		Oxidative Phosphorylation	pyrophosphate (P	GC/MS	2078	C00013	HMDB00250	0.94	1.11	0.68	0.80			
929			phosphate	GC/MS	11438	C00009	HMDB01429	0.87	0.80	1.07	0.99			
2064		Purine Metabolsim	adenosine	LC/MS pos	555	C00212	HMDB00050	0.87	1.26	0.69	1.00			
2065			adenine	GC/MS	554	C00147	HMDB00034	0.63	1.75	0.36	1.01			
2095		Purine Metabolism, Guanine containing	guanosine	LC/MS pos	1573	C00387	HMDB00133	0.81	5.53	0.14	0.97			
2096			guanine	LC/MS pos	32352	C00242	HMDB00132	0.79	0.97	0.82	1.01			
2127	Nucleotide	Pyrimidine Metabolism, Uracil-Contain	uridine	LC/MS neg	606	C00299	HMDB00296	1.73	8.09	0.20	0.93			
2128			uracil	GC/MS	605	C00106	HMDB00300	1.71	5.72	0.33	1.09			
2149		Pyrimidine Metabolism, Cytidine conta	cytidine 5'-monopl	LC/MS pos	2372	C00055	HMDB00095	1.08	3.69	0.27	0.93			
2152			cytidine	LC/MS pos	514	C00475	HMDB00089	0.71	0.80	0.79	0.90			
2168		Pyrimidine Metabolism, Thymine conta	thymidine	LC/MS neg	39539	C00214	HMDB00273	0.87	4.14	0.16	0.77			
2169			thymine	GC/MS	604	C00178	HMDB00262	0.82	1.57	0.43	0.82			

Statistical Values

Pathway Sort Order	ANOVA Contrasts: (+)GLU(+)GLCN / (+)GLU(-)GLCN *p*-value	*q*-value	(-)GLU(+)GLCN / (-)GLU(-)GLCN *p*-value	*q*-value	(-)GLU(-)GLCN / (+)GLU(-)GLCN *p*-value	*q*-value	(-)GLU(+)GLCN / (+)GLU(+)GLCN *p*-value	*q*-value	Two-Way ANOVA: Glucose Main Effect *p*-value	*q*-value	Glucosamine Main Effect *p*-value	*q*-value	Glucose:Glucosamine Interaction *p*-value	*q*-value
36	0.0102	0.0185	0.5379	0.1213	0.0000	0.0000	0.0079	0.1481	0.0000	0.0000	0.1338	0.0499	0.0237	0.0136
37	0.0396	0.0473	0.3661	0.0900	0.0006	0.0002	0.0115	0.1829	0.0001	0.0001	0.0389	0.0196	0.3773	0.1368
296	0.0229	0.0337	0.3067	0.0791	0.5537	0.0929	0.4260	0.9963	0.8833	0.2773	0.0220	0.0118	0.3290	0.1223
298	0.0068	0.0151	0.0025	0.0011	0.0000	0.0000	0.0325	0.3352	0.0000	0.0000	0.7632	0.1996	0.0002	0.0002
299	0.0359	0.0462	0.0400	0.0135	0.0000	0.0000	0.0958	0.6742	0.0000	0.0000	0.9706	0.2365	0.0051	0.0035
731	0.4002	0.2321	0.0000	0.0000	0.0000	0.0000	0.7465	0.9963	0.0000	0.0000	0.0000	0.0000	0.0000	0.0000
732	0.4533	0.2465	0.0003	0.0002	0.0000	0.0000	0.0583	0.4807	0.0000	0.0000	0.0017	0.0014	0.0197	0.0118
735	0.9906	0.4024	0.0000	0.0000	0.0000	0.0000	0.7827	0.9963	0.0000	0.0000	0.0000	0.0000	0.0000	0.0000
742	0.6859	0.3212	0.0035	0.0014	0.0001	0.0000	0.0655	0.5188	0.0001	0.0001	0.0160	0.0090	0.0536	0.0271
748	0.8559	0.3711	0.0127	0.0047	0.0001	0.0000	0.0237	0.3047	0.0000	0.0000	0.0522	0.0246	0.0863	0.0415
751	0.0937	0.0891	0.0829	0.0247	0.0043	0.0012	0.0050	0.1463	0.0002	0.0002	0.0197	0.0107	0.9633	0.2760
756	0.1648	0.1321	0.0937	0.0270	0.1486	0.0290	0.2500	0.9902	0.0719	0.0338	0.0348	0.0178	0.8245	0.2458
759	0.1124	0.1033	0.0012	0.0006	0.0046	0.0012	0.2854	0.9963	0.0066	0.0040	0.0010	0.0010	0.1543	0.0670
765	0.2384	0.1735	0.0000	0.0000	0.0000	0.0000	0.9650	0.9963	0.0000	0.0000	0.0000	0.0000	0.0000	0.0001
873	0.0353	0.0462	0.0001	0.0001	1.0000	0.1514	0.0232	0.3047	0.0975	0.0448	0.0001	0.0001	0.0975	0.0451
883	0.0000	0.0002	0.0000	0.0000	0.0000	0.0000	0.6864	0.9963	0.0000	0.0000	0.0000	0.0000	0.0000	0.0000
908	0.7077	0.3290	0.0000	0.0000	0.0000	0.0000	0.0067	0.1481	0.0000	0.0000	0.0000	0.0000	0.0000	0.0000
917	0.5002	0.2649	0.0584	0.0186	0.0033	0.0009	0.5284	0.9963	0.0108	0.0062	0.3614	0.1163	0.0713	0.0355
918	0.4009	0.2321	0.0000	0.0000	0.0000	0.0000	0.0007	0.0354	0.0000	0.0000	0.0005	0.0005	0.0000	0.0001
928	0.7133	0.3298	0.8739	0.1722	0.2559	0.0475	0.5318	0.9963	0.2162	0.0861	0.8824	0.2192	0.7100	0.2286
929	0.3089	0.1973	0.0864	0.0256	0.7187	0.1142	0.6978	0.9963	0.9841	0.3005	0.0577	0.0264	0.5973	0.2004
2064	0.2973	0.1959	0.1353	0.0368	0.0084	0.0022	0.7692	0.9963	0.0339	0.0182	0.7347	0.1952	0.0781	0.0382
2065	0.0049	0.0125	0.0013	0.0006	0.0000	0.0000	0.7920	0.9963	0.0000	0.0000	0.6832	0.1860	0.0001	0.0001
2095	0.0418	0.0492	0.0000	0.0000	0.0000	0.0000	0.5778	0.9963	0.0000	0.0000	0.0000	0.0000	0.0000	0.0000
2096	0.0310	0.0411	0.8189	0.1634	0.0467	0.0100	0.9760	0.9963	0.1441	0.0625	0.0861	0.0362	0.1552	0.0670
2127	0.0013	0.0057	0.0000	0.0000	0.0000	0.0000	0.4491	0.9963	0.0000	0.0000	0.0000	0.0000	0.0000	0.0000
2128	0.0001	0.0006	0.0000	0.0000	0.0000	0.0000	0.4910	0.9963	0.0000	0.0000	0.0000	0.0000	0.0000	0.0000
2149	0.6095	0.2915	0.0000	0.0000	0.0000	0.0000	0.5856	0.9963	0.0000	0.0000	0.0000	0.0000	0.0000	0.0001
2152	0.0028	0.0084	0.0446	0.0146	0.0229	0.0055	0.2461	0.9902	0.0176	0.0099	0.0008	0.0008	0.3799	0.1369
2168	0.2614	0.1808	0.0000	0.0000	0.0000	0.0000	0.0285	0.3258	0.0000	0.0000	0.0000	0.0000	0.0000	0.0000
2169	0.3150	0.1973	0.0434	0.0144	0.0001	0.0000	0.1229	0.7035	0.0002	0.0002	0.4353	0.1350	0.0356	0.0192

Supplementary Table 3 (Related to Methods)

Immunoblot Antibodies

Antibody	Dilution	Species	Source
Anti-GLUT1	1:500	Mouse	Abcam (ab40084)
Anti-GLUT2	1:1000	Rabbit	Santa Cruz Biotechnology (sc-9117)
Anti-GLUT3	1:1000	Mouse	Santa Cruz Biotechnology (sc-74399)
Anti-PCNA	1:2000	Mouse	Santa Cruz Biotechnology (sc-56)
Anti-β-ACTIN	1:5000	Mouse	Sigma (A5441)
Anti-HK1	1:500	Rabbit	Cell Signaling Technology (2024)
Anti-HK2	1:500	Rabbit	Cell Signaling Technology (2867)
Anti-PKM1/2	1:500	Rabbit	Cell Signaling Technology (3190)
Anti-PKM2	1:500	Rabbit	Cell Signaling Technology (4053)
Anti-PFKP	1:500	Rabbit	Cell Signaling Technology (8164)
Anti-PDHA	1:500	Rabbit	Cell Signaling Technology (3205)
Anti-LDHA	1:500	Rabbit	Cell Signaling Technology (3582)
Anti-GlcNAc	1:1000	Mouse	Santa Cruz Biotechnology (sc-59624)
Anti-OXPHOS cocktail	1:500	Mouse	Abcam (ab110413)
Anti-NANOG	1:1000	Rabbit	Cell Signaling Technology (8822)
Anti-SOX2	1:1000	Rabbit	Cell Signaling Technology (2748)
Anti-OCT4	1:1000	Rabbit	Santa Cruz Biotechnology (sc-9081)
Anti-Mouse IgG (HRP-coupled)	1:3000	Goat	Santa Cruz Biotechnology (sc-2055)
Anti-Rabbit IgG (HRP-coupled)	1:3000	Donkey	GE Healthcare (NA934V)

Supplementary Table 4 (Related to Experimental Procedures)

Immunoprecipitation antibodies

Antibody	Amount	Species	Source
Anti-OCT4	1 μg	Rabbit	Santa Cruz Biotechnology (sc-9081)
Anti-NANOG	1:100 (vol:vol)	Rabbit	Cell Signaling Technology (8822)
Anti-SOX2	1:100 (vol:vol)	Rabbit	Cell Signaling Technology (2748)
Anti-GlcNAc	1 μg	Mouse	Santa Cruz Biotechnology (sc-59624)
Nonimmune Mouse IgG	1 μg	Mouse	Santa Cruz Biotechnology (sc-2025)
Nonimmune Rabbit IgG	1 μg	Rabbit	Calbiochem (12-370)

SUPPLEMENTARY METHODS

LG-ESC Culture and Transfection

Murine LG-ESC were grown in low glucose DMEM (Life Technologies) with 15% fetal calf serum (Atlanta Biologicals) as described[1] $\pm$ GlcN (Sigma), added at 0.8 mM unless otherwise indicated. To maintain glucose concentrations at 5.5 mM in cultures lasting more than 2 days, media glucose concentrations were measured with a blood glucose meter (Roche Diagnostics) and then media were brought to 5.5 mM by adding appropriate volumes of 25% glucose as described[2]. Cultures are tested for mycoplasma contamination using the MycoAlert Mycoplasma Detection Kit (Lonza).

G2KD-LG-ESC were generated by stably transfecting LG-ESC with pSUPER (OligoEngine) containing a short hairpin RNA (shRNA) against *Glut2/Slc2a2*[3] as described[4]. A control cell line, C-LG-ESC, was generated by transfecting with empty pSUPER vector. OGT-KD-LG-ESC cells were derived by stably transfecting LG-ESC with doxycycline (Dox)-inducible pSingle (Clontech) containing one of three shRNA sequences against *Ogt* mRNA that were previously reported[5] or a scrambled shRNA sequence (Sc-LG-ESC)[6]. shRNA expression was induced with 1 µg/ml Dox). 1-10 mM alloxan (Sigma) was added to LG-ESC cultures to inhibit OGT enzyme activity.

LG-ESC Sex Determination

The sex of each cell line was determined by performing PCR of genomic DNA for *Zfy*, a Y chromosome marker as described[7,8] except *Glut2* as an autosomal PCR control[9].

^{3}H-GlcN Transport Assay

3 x 10^5 cells were cultured in 35 mm dishes for 48 hr in 5.5 mM glucose media. Cells were incubated with 10 µCi D-[6-^{3}H(N)]GlcN (PerkinElmer) in 0.8 mM GlcN with 5 mM or 16 mM 2-deoxy-D-glucose for 20 min. Reactions were stopped and cells were solubilized as described[10]. 950 µl of cell lysate were counted using a liquid scintillation counter. 50 µl of cell lysate were used to measure protein concentration with Protein Assay Dye Reagent (Bio-Rad).

2-deoxy-D-Glucose Transport Assay

3 x 10^5 cells were cultured in 35 mm dishes for 48 hr in 5.5 mM glucose media, then were incubated with 0.125 mM or 0.4 mM of the fluorescent 2-deoxy-D-glucose analog, 2-NBD-glucose (2-deoxy-2-[(7-nitro-2,1,3- benzoxadiazol-4-yl)amino]-D-glucose, Cayman Chemical) in 5 mM or 16 mM 2-deoxy-D-glucose, respectively, ± 0.8 mM GlcN, for 20 min. Reactions were stopped by solubilizing cells according to the manufacturer's recommendations. 485/535 nm (excitation/emission) was measured using 50 µl of cell lysate in a 96 well plate. Protein was assayed as above.

Real Time RT-PCR

Total RNA was extracted after 4 days of culture using Ultraspec reagent (Biotecx Laboratories, Friendswood, TX) and 200 ng were reverse transcribed using the High-Capacity cDNA Reverse Transcription Kit (Life Technologies). Real time PCR was performed using TaqMan PCR Master Mix (Life Technologies) as described[11]. Primers and FAM-labeled probes for *Nanog* (Mm 02384862), *Sox2* (Mm 00488369), *Oct4* (Mm

00658129), and VIC-labeled probe for *rRNA* (4310893E) were obtained from Life Technologies.

Extracellular Flux (XF) Analysis

3 x 10^3 cells were cultured for 12 hr then were washed with DMEM (0 glucose) with 143 mM NaCL and incubated for 1 hr in DMEM (5.5 mM glucose with 2 mM glutamine) $\pm$ 0.8 mM GlcN in a 37° oven. Extracellular acidification rates (ECAR) and oxygen consumption rates (OCR) were measured using a Seahorse Extracellular Flux Analyzer (XF24) 6 times over 1.5 hr. DNA was extracted with 50 mM NaOH heated to 95 °C for 30 min followed by neutralization with 1M Tris-Cl pH 6.8. DNA concentrations were measured by NanoDrop™ (Thermo Scientific).

Metabolic Profiling

Six replicate 10 cm culture dishes of 5 x 10^6 cells were grown under 4 culture conditions ((+)GLU(-)GLCN; (+)GLU(+)GLCN; (-)GLU(-)GLCN; (+)GLU(+)GLCN), in which cultures were grown in 5.5 mM glucose $\pm$ 0.8 mM GlcN for 47 hr, and then media were replaced with the same media, or 0 glucose media $\pm$ GlcN, for the final h of culture. Cells were harvested by scraping into PBS, pelleted, flash frozen in liquid nitrogen, and stored at -80 °C until analysis by Metabolon, Inc. Three independent platforms were employed for untargeted metabolic profiling: ultrahigh performance liquid chromatography/tandem mass spectrometry (UHLC/MS/MS) optimized for basic species, UHLC/MS/MS optimized for acidic species, and gas chromatography/mass spectrometry (GC/MS). Samples were processed essentially as described previously[12,13]. For UHLC/MS/MS

analysis, aliquots were separated using a Waters Acquity UPLC and analyzed using an LTQ mass spectrometer (Thermo Fisher Scientific, Inc.), which consisted of an electrospray ionization (ESI) source and linear ion-trap (LIT) mass analyzer. The MS instrument scanned 99-1000 *m/z* and alternated between MS and MS^2 scans using dynamic exclusion with approximately 6 scans per second. Derivatized samples for GC/MS were separated on a 5% phenyldimethyl silicone column with helium as the carrier gas and a temperature ramp from 60°C to 340°C and then analyzed on a Thermo-Finnigan Trace DSQ MS (Thermo Fisher Scientific, Inc.) operated at unit mass resolving power with electron impact ionization and a 50-750 atomic mass unit scan range. Metabolites were identified by automated comparison of the ion features in the experimental samples to a reference library of chemical standard entries that included retention time, molecular weight (*m/z*), preferred adducts, and in-source fragments as well as associated MS spectra and curated by visual inspection for quality control using software developed at Metabolon[14]. Experimental samples and controls were randomized across a one-day platform run. Any missing values were assumed to be below the limits of detection and for statistical analyses and data display purposes, these values were imputed with the compound minimum (minimum value imputation). Statistical analysis of log-transformed data was performed using "R" (http://cran.r-project.org/). Two-way ANOVA was performed to determine main effects of glucose and GlcN and glucose:GlcN interaction. Student t-test was performed to compare data between experimental groups. Multiple comparisons were accounted for by estimating the false discovery rate (FDR) using q-values[15].

SUPPLEMENTAL REFERENCES

1 Jung, J. H., Wang, X. D. & Loeken, M. R. Mouse embryonic stem cells established in physiological-glucose media express the high KM Glut2 glucose transporter expressed by normal embryos. *Stem cells translational medicine* **2**, 929-934, doi:10.5966/sctm.2013-0093 (2013).

2 Sanders, K., Jung, J. H. & Loeken, M. R. Use of a murine embryonic stem cell line that is sensitive to high glucose environment to model neural tube development in diabetic pregnancy. *Birth defects research. Part A, Clinical and molecular teratology* **100**, 584-591, doi:10.1002/bdra.23281 (2014).

3 Bain, J. R., Schisler, J. C., Takeuchi, K., Newgard, C. B. & Becker, T. C. An adenovirus vector for efficient RNA interference-mediated suppression of target genes in insulinoma cells and pancreatic islets of langerhans. *Diabetes* **53**, 2190-2194 (2004).

4 Wang, X. D., Morgan, S. C. & Loeken, M. R. Pax3 stimulates p53 ubiquitination and degradation independent of transcription. *PLoS One* **6**, e29379, doi:10.1371/journal.pone.0029379 (2011).

5 Jang, H. *et al.* O-GlcNAc regulates pluripotency and reprogramming by directly acting on core components of the pluripotency network. *Cell Stem Cell* **11**, 62-74, doi:10.1016/j.stem.2012.03.001 (2012).

6 Wei, D. & Loeken, M. R. Increased DNA methyltransferase 3b (dnmt3b)-mediated CpG island methylation stimulated by oxidative stress inhibits expression of a gene required for neural tube and neural crest development in diabetic pregnancy. *Diabetes* **63**, 3512-3522, doi:10.2337/db14-0231 (2014).

7 Pani, L., Horal, M. & Loeken, M. R. Rescue of neural tube defects in Pax-3-deficient embryos by p53 loss of function: implications for Pax-3- dependent development and tumorigenesis. *Genes Dev* **16**, 676-680, doi:10.1101/gad.969302 (2002).

8 Sah, V. P. *et al.* A subset of p53-deficient embryos exhibit exencephaly. *Nat Genet* **10**, 175-180 (1995).

9 Guillam, M.-T. *et al.* Early diabetes and abnormal postnatal pancreatic islet development in mice lacking Glut-2. *Nature Genetics* **17**, 327-330 (1997).

10 Uldry, M., Ibberson, M., Hosokawa, M. & Thorens, B. GLUT2 is a high affinity glucosamine transporter. *FEBS Lett* **524**, 199-203 (2002).

11 Chang, T. I. *et al.* Oxidant regulation of gene expression and neural tube development: Insights gained from diabetic pregnancy on molecular causes of neural tube defects. *Diabetologia* **46**, 538-545 (2003).

12 Lawton, K. A. *et al.* Analysis of the adult human plasma metabolome. *Pharmacogenomics* **9**, 383-397, doi:10.2217/14622416.9.4.383 (2008).

13 Sreekumar, A. *et al.* Metabolomic profiles delineate potential role for sarcosine in prostate cancer progression. *Nature* **457**, 910-914, doi:10.1038/nature07762 (2009).

14 Dehaven, C. D., Evans, A. M., Dai, H. & Lawton, K. A. Organization of GC/MS and LC/MS metabolomics data into chemical libraries. *Journal of cheminformatics* **2**, 9, doi:10.1186/1758-2946-2-9 (2010).

15 Storey, J. D. & Tibshirani, R. Statistical significance for genomewide studies. *Proc Natl Acad Sci U S A* **100**, 9440-9445, doi:10.1073/pnas.1530509100 (2003).
